# Autoimmune Type 1 Diabetes in the Era of Disease‐Modifying Immune Therapy

**DOI:** 10.1002/dmrr.70091

**Published:** 2025-10-09

**Authors:** Aaron W. Michels, Peter A. Gottlieb, Bryce Nelson, Colin Dayan

**Affiliations:** ^1^ Barbara Davis Center for Diabetes University of Colorado Anschutz Medical Campus Aurora Colorado USA; ^2^ Children's Hospital of Richmond Virginia Commonwealth University Richmond Virginia USA; ^3^ Cardiff University Cardiff UK

**Keywords:** autoimmune, disease‐modifying therapy, progression, screening, teplizumab, type 1 diabetes

## Abstract

Disease‐modifying therapies have been used to treat the underlying causes of autoimmune diseases for over half a century. However, until recently, type 1 diabetes (T1D), the autoimmune form of diabetes, had not entered this therapeutic landscape. The approval of teplizumab, an anti‐CD3 monoclonal antibody and the first disease‐modifying therapy for use in individuals with preclinical T1D, has caused a major shift in the way healthcare providers can treat the T1D disease course. In this review, we discuss the chronic autoimmune nature of T1D and provide an overview of disease‐modifying therapies that are under investigation to target the autoimmune mechanisms in T1D to preserve residual beta‐cell function and prevent disease progression. The considerations for implementing these therapies into clinical practice are also discussed.

## Graphical abstract

While disease‐modifying therapies (DMTs) have been used to treat autoimmune diseases for decades, until recently, DMTs had not been used to treat autoimmune type 1 diabetes (T1D). The approval of the first DMT for use in individuals with preclinical T1D, teplizumab, has ushered in a new era in which healthcare providers can slow the T1D disease course. This review discusses the chronic, irreversible, autoimmune nature of T1D, provides an overview of DMTs under investigation to target autoimmune mechanisms in T1D to preserve residual beta‐cell function and prevent disease progression, and provides considerations for implementing these therapies in clinical practice.

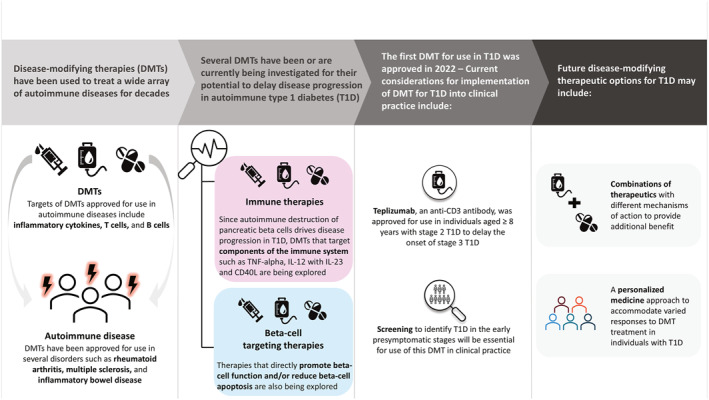

## 1 Introduction

Over the past 60 years, autoimmune disorders and those of immune dysregulation have moved away from just the treatment of symptoms to the use of disease‐modifying therapies (DMTs) to alter the course of disease [1, 2]. For most of that period, the field of type 1 diabetes (T1D) had not entered this era of immune therapies or biological agents to treat the underlying autoimmune pathology. However, clinical approval of the first DMT and multiple clinical trials have demonstrated the efficacy and safety of immune therapies for T1D. This has shifted how treatment of this disease can be approached and led to a delay in the need for insulin treatment [3, 4, 5, 6, 7]. This review will provide a brief overview of the stages of T1D development and a description of teplizumab now approved for clinical use to delay the onset of clinical diabetes and those therapies under investigation to treat various stages of T1D. Important considerations for the implementation of these novel therapeutics in clinical practice, and the implications of treating the underlying immunology of T1D for the field, will be discussed as the focus of this review.

## 2 T1D Is a Chronic, Progressive Autoimmune Disease

T1D is a chronic autoimmune disease that progresses in stages, ultimately resulting in insulin deficiency and elevated blood glucose levels [8, 9]. Although the exact cause of T1D is unknown, studies have shown that genetics combined with environmental factors may trigger immune‐related mechanisms associated with the development of pancreatic beta‐cell autoimmunity [10, 11].

An individual's genetic profile plays an important role in determining whether they develop autoimmune diseases, including T1D [11]. Many autoimmune diseases have similar genetic backgrounds that predispose to immune dysregulation [12, 13]. As a result, individuals with one autoimmune disease are more likely to develop others [14]. The clinical diagnosis of T1D has been shown to typically precede coeliac disease and autoimmune thyroid disease. This may be because of the more subtle onset and less recognisable clinical features at presentation of these other disorders as well as differences in average age at presentation of autoimmune disorders [15, 16, 17]. Additionally, individuals with preexisting coeliac disease or autoimmune thyroid diseases have been shown to have an increased risk of developing T1D [14, 18]. Genetic factors shown to influence an individual's susceptibility for T1D include human leucocyte antigen (HLA) genes, specifically HLA class II genes, which are estimated to be responsible for approximately 50% of an individual's genetic risk for T1D [12, 13, 19, 20, 21]. Non‐HLA genes have also been demonstrated to confer susceptibility to T1D [13]. Regardless of these strong associations between genetics and disease, most individuals with diabetes‐susceptible HLA class II genes do not develop T1D, and more broadly, countries with similar genetic backgrounds can have variable T1D rates [19, 22]. Additionally, recent data demonstrated a weaker familial influence and lower heritability for adult‐onset T1D compared with childhood‐onset T1D. Together, these data suggest that nongenetic factors, including environmental factors, also play an important role in the clinical manifestation of disease, especially in adults [11, 19, 23].

**FIGURE 1:**
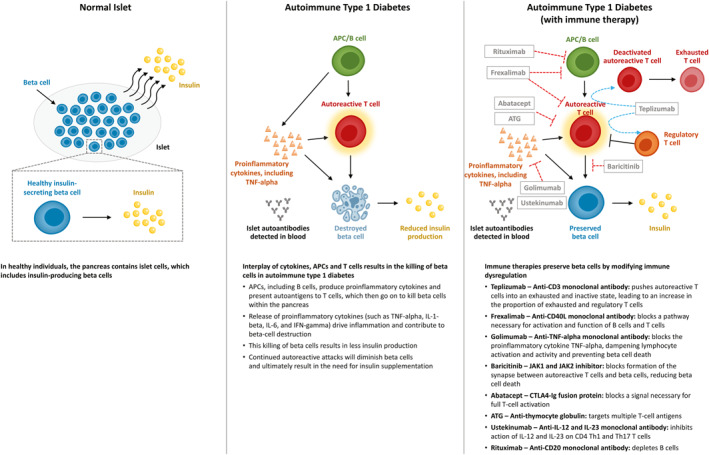
Autoimmune pathways in T1D and immune therapeutic approaches that modulate disease. APC, antigen‐presenting cell; CD40L, CD40 ligand; CTLA4, cytotoxic T‐lymphocyte associated protein 4; IFN‐gamma, interferon gamma; Ig, immunoglobulin; IL‐1‐beta, interleukin 1‐beta; IL‐6, interleukin 6; IL‐12, interleukin 12; IL‐23, interleukin 23; JAK1/2, Janus kinase 1/2; TNF‐alpha, tumour necrosis factor alpha.

In healthy individuals, beta cells within the pancreatic islets secrete enough insulin to allow the body to maintain normal blood glucose levels in response to daily activities [24]. They also have immune self‐tolerance. In individuals with T1D, there is a reduction in the amount of functional beta‐cell mass, resulting in impaired insulin production [21, 24]. This loss of beta‐cell mass is the result of a loss of immunological self‐tolerance that allows autoreactive T cells that recognise self‐antigens within the pancreatic islets to escape thymic selection and contribute to the destruction of beta cells [3, 25, 26, 27] (Figure 1). However, reduction in pancreas size during T1D is likely not due to islet‐directed autoimmunity as islets comprise only a small percentage of pancreas volume, although mechanisms underlying this process are poorly understood [28]. Other immune mediators, such as B cells, have also been shown to be involved in T1D disease progression by acting as antigen‐presenting cells presenting self‐antigens derived from islets to T cells [29]. Pro‐inflammatory cytokines also contribute to the diabetogenic process directly by causing beta‐cell injury and indirectly by enhancing inflammation and promoting lymphocyte activation and activity [6] (Figure 1). During this process, the immune system is dysregulated, and both innate and adaptive immune mechanisms are hyperactive [30]. Therefore, many DMTs that target disease progression in T1D are immune therapies that aim to dampen this response.

It is important to note that islet autoimmunity precedes clinical manifestations of T1D (i.e., sustained hyperglycemia), often by several years [10, 21, 31]. Accordingly, the disease process of T1D has been divided into stages based on the detection of islet autoimmunity by measuring islet autoantibodies in peripheral blood and the level of glycaemia an individual experiences. Four biochemical autoantibodies have been well validated as predictors of T1D risk and disease progression: (1) islet autoantibodies that recognise insulin (IAA), (2) glutamic acid decarboxylase (GAD), (3) protein tyrosine phosphatase‐like IA‐2 (IA‐2) and (4) zinc transporter 8 (ZnT8) [32]. Although islet autoantibody detection is the main method used in the clinic to identify islet autoimmunity, autoreactive T cells are also detectable in these individuals in laboratory settings [27].

The stages of T1D represent increasing levels of beta‐cell destruction and worsening progressive dysglycemia. Stage 1 T1D is defined by the presence of 2 or more islet autoantibodies with normal blood glucose levels (normoglycemia) [21, 33]. While this stage is asymptomatic, the lifetime risk of developing symptomatic T1D after reaching this stage in childhood or adolescence approaches nearly 100% [21, 34]. Stage 2 is defined by the presence of 2 or more islet autoantibodies and the development of impaired glucose tolerance (dysglycemia) [21, 33]. Lastly, stage 3 T1D is defined by the onset of clinical symptoms typically associated with diabetes, such as polyuria and polydipsia, as well as sustained hyperglycemia in the established ranges diagnostic of clinical diabetes [21, 33]. In short, the degree of dysglycemia differs from stage 1 to 3 T1D, with normoglycemia at stage 1 and sustained hyperglycemia at stage 3. Classification of individuals with one islet antibody is also dependent on the degree of dysglycemia experienced by the individual [34]. In the absence of dysglycemia, these individuals are considered to be at low risk of clinical T1D and can be monitored accordingly. However, the presence of one or more autoantibodies with persistent hyperglycemia satisfies the diagnostic criteria for stage 3 T1D [35].

Individuals with one islet autoantibody have a low risk of progression to clinical T1D, with 10% of these children developing clinical diabetes in one study. However, most children with multiple islet autoantibodies will progress to clinical T1D within 15 years. Specifically, it has been shown that 69.7% of children with multiple islet antibodies developed stage 3 T1D within 10 years, and 84.2% developed clinical disease within 15 years [34]. The increased beta‐cell dysfunction that occurs as T1D progresses is permanent [22].

Progressive beta‐cell loss and the subsequent development of clinical T1D have many negative short‐ and long‐term effects. Poorly managed T1D can cause increased adverse glycaemic events, including hypoglycemia, hyperglycemia, and life‐threatening diabetic ketoacidosis (DKA) [36, 37]. Severe hypoglycemia can cause neurological consequences, such as seizures, brain damage, and intellectual impairment. Hyperglycemia, on the other hand, is a known risk factor for macro‐ and microvascular complications, including cardiovascular diseases, neuropathy, nephropathy and retinopathy [36]. To manage the loss of metabolic control associated with the disease, individuals require daily exogenous insulin and frequent glucose monitoring for the rest of their lives [21, 36].

The rate of progression in the early stages of disease—from islet autoimmunity to the onset of clinical disease—is influenced by a number of factors. Age at islet autoantibody seroconversion, for example, has been shown to correlate with the rate of progression to stage 3 T1D, with younger individuals progressing at a faster rate [32, 38]. The number and levels of specific types of islet autoantibodies can also influence the rate of disease progression [32]. IA‐2A and ZnT8A positivity, either on their own or in combination with other islet autoantibody positivity, have generally been associated with higher rates of progression [39, 40]. On the other hand, IAA and GAD positivity were more commonly associated with lower rates of progression [32]. Children with lower initial levels of IAA experienced slower progression to clinical diabetes [41]. Moreover, autoantibody profiles characterised by a combination of various factors, specifically the age at seroconversion and the sequence of appearance of IAA, GAD and IA‐2A, have been shown to be predictive of progression rates. Children diagnosed at a younger age who had an initial appearance of IAA, followed by GAD and IA‐2A, had the highest progression rates to clinical diabetes in one study [42].

The risk of progression to clinical T1D has been shown to vary based on the stringency of how multiple islet autoantibodies are defined. Individuals with multiple islet autoantibodies under the most stringent conditions (2 or more islet autoantibodies positive during the same visit with 2 or more antibodies persistent at a subsequent visit) had a higher risk of progression than individuals with multiple islet autoantibodies under less stringent conditions (positivity for 2 islet autoantibodies occurring separately at different time points or not persistent) [38]. Genetic factors, such as non‐HLA T1D susceptibility genes, including insulin variable number of tandem repeats, interleukin 2 (IL‐2), CD25, and protein tyrosine phosphatase non‐receptor type 22, have also been demonstrated to influence disease progression [13, 21]. Additionally, higher T1D genetic risk scores (GRSs), which incorporate HLA and non‐HLA T1D‐associated nucleotide polymorphisms, have been associated with increased rates of disease progression in patients with single‐autoantibody positivity [43]. GRS has since been improved (GRS2) and is now an even better predictor of T1D development in the general population [44, 45].

Age has an impact on how these risk factors influence disease progression. Analysis of longitudinal data has demonstrated that in individuals with specific high‐risk HLA alleles, the hazard ratio for progression from multiple autoantibody positivity to clinical T1D decreased with increasing age [46]. In the same study, the effect of the presence of islet autoantibodies GAD or IAA on the risk of progression from single autoantibody positivity to multiple autoantibody positivity or clinical T1D also varied with age. Specifically, in individuals with GAD autoantibodies, the risk of progression increased with increasing age of the individual, whereas in individuals with IAA autoantibodies, the risk of progression decreased with increasing age of the individual [46]. Additionally, the ability of the T1D GRS to predict disease progression from single‐autoantibody positivity to multiple‐autoantibody positivity was significant only in individuals under age 35 [43].

It is important to note that studies exploring the rate of T1D progression have mainly been conducted in paediatric populations [32]. Not much is known about the rate of progression in adult onset T1D [47, 48, 49]. Additional studies in older cohorts are therefore crucial, not just to gain a deeper understanding of disease progression but also to stratify these individuals for risk of developing disease, as well as their ability to respond to DMTs.

## 3 The Current Therapeutic Approach to T1D

The current approach to clinical T1D treatment, lifelong replacement of insulin, has been the standard of care for over 100 years; however, a high burden of disease still exists [50, 51]. Up to 1 in 4 people with clinical diabetes who are prescribed insulin report cost‐related insulin underuse. Insulin doses also need to be routinely adjusted, which can be challenging for patients and families [52]. Importantly, insulin replacement has no effect on the autoimmune disease process that drives T1D development [50]. Although the use of diabetes technology with automated insulin delivery systems can help individuals achieve excellent glycaemic levels, these systems have no impact on the preservation of stimulated C‐peptide levels—a stable measure of endogenous beta‐cell function [53, 54]. However, several DMTs have been shown to preserve beta‐cell function by slowing the rate of C‐peptide decline when administered to individuals with newly diagnosed T1D.

## 4 Screening for Presymptomatic T1D: Importance and Considerations for Implementation

Advances in the development of novel therapies, which can modify the T1D disease process by preserving pancreatic beta‐cell function, have made the detection of presymptomatic T1D of utmost importance. Earlier recognition and treatment can now delay symptomatic disease and improve outcomes in individuals with the disease [4]. Currently, T1D is mostly diagnosed at the symptomatic stage, when the individual has already experienced significant beta‐cell loss, can be critically ill, and requires exogenous insulin [21, 55].

Earlier diagnosis—before significant beta‐cell loss, when patients are in stage 1 or stage 2 T1D—would allow individuals to start therapy that could delay disease progression. It would also allow for monitoring to avoid becoming part of the large percentage of patients who become acutely ill and present with life‐threatening DKA at the time of diagnosis. Not only is DKA life threatening but it also predicts a higher risk of subsequent all‐cause mortality, major cardiovascular events, advanced kidney disease, advanced neuropathy, severe hypoglycemia, and recurrent DKA [56]. Furthermore, DKA at clinical diagnosis has been associated with worse long‐term glycaemic control and an increased risk of cognitive impairment [57, 58]. DKA has also been shown to cause psychological distress in children and their caregivers and put a substantial economic burden on individuals with T1D and healthcare systems [58, 59]. DKA rates at the time of diagnosis for clinical T1D are still as high as 60% in the United States (US) [60]. Studies have demonstrated that early identification and regular monitoring of patients at risk can reduce DKA rates at the time of diagnosis to less than 5% of patients [61, 62]. Reductions in DKA and associated complications may make screening cost‐effective in populations and regions with high rates of DKA [63].

Because of the need to reduce the risk of DKA at diagnosis, coupled with advances that now enable individuals to delay clinical onset of T1D with immune therapy, a renewed focus should be placed on early identification and screening. Screening children for islet autoantibodies would facilitate the detection of T1D at the earliest disease stages [55]. Autoantibodies, as markers of T1D, may develop before 1 year of age but can also appear later in childhood. Thus, autoantibody screening may need to occur at multiple timepoints throughout childhood [64].

The affinity level of islet autoantibodies also influences an individual's risk of developing clinical T1D. The presence of lower‐affinity single islet autoantibodies has been associated with no increased risk of developing clinical T1D, and these autoantibodies tend to be lost over time. Nonetheless, radiobinding assays that are widely used to detect islet autoantibodies can detect these low‐affinity autoantibodies, resulting in false‐positive results [40]. Therefore, confirmation of single antibody positivity in a separate test by using newer assays that detect only high‐affinity autoantibodies that are more predictive of clinical T1D development may increase the likelihood of identifying individuals at risk [65].

Most T1D screening efforts have focused on screening relatives of individuals with clinical T1D because relatives have an approximate 15‐fold increase in risk compared with those without a relative with T1D [64]. While this group should be screened, approximately 80%–85% of those who will be diagnosed with T1D do not have a family history, highlighting the narrow approach of screening those with a family history [66]. This may lead to a reduced ability to influence the severity of the disease at diagnosis, including DKA, and the opportunity to preserve beta cells with DMTs.

Screening programs for early T1D in the general population have been suggested as the long‐term goal [64]. But until they are more widely implemented, healthcare providers outside structured screening programs will bear the responsibility of screening high‐risk individuals [64]. Those considered at high risk of T1D development could be expanded beyond those with first‐degree relatives to include individuals with other autoimmune conditions. As previously mentioned, the presence of one autoimmune disease may confer increased risk for the subsequent development of T1D. There is robust evidence suggesting that the presence of coeliac disease, autoimmune thyroid disease, Addison's disease, and juvenile idiopathic arthritis makes individuals high‐risk for development of T1D, particularly for coeliac disease and thyroid disease [14, 67]. Indeed, the American Diabetes Association Professional Practice Committee Roundtable Report recommended screening for T1D in children and adolescents with autoimmune thyroid or coeliac disease [68]. However, the red flag in clinical practice to screen for presymptomatic T1D could come from the presence of any autoimmune disease, since there is varying evidence for many additional diseases conferring high risk for development of T1D due to shared genetic susceptibility and predisposition [67]. The co‐occurrence of autoimmune diseases has been well documented, with 25% of individuals with an autoimmune disease expected to develop additional autoimmune disorders [69]. Nonetheless, a more practical course of action for clinics/institutions with limited resources for screening could be to take a risk stratification approach in which individuals with relatives with T1D are given highest priority for screening, followed by individuals with a personal or family history of other autoimmune diseases, and then lastly, individuals in the general population at specified ages or concurrently with other routine screening tests.

Some studies have demonstrated that screening, particularly receiving a positive autoantibody result, is associated with a risk of negative psychological impact or anxiety; however, caregivers of children diagnosed with presymptomatic T1D reported experiencing less stress at metabolic staging compared with caregivers of children diagnosed with clinical T1D without prior screening [62, 64]. Therefore, with appropriate education and care, islet autoantibody screening is likely to result in less psychological stress anticipating and preparing for the clinical diagnosis compared with abruptly discovering one has a disease that affects day‐to‐day life through the development of clinical symptoms, typically in an emergency setting. Understanding the implications of positive screens on the risk of clinical progression and why early detection is vital to outcomes has been shown to relieve psychological stress. Healthcare providers may want to emphasise the value of early detection and the potential for prevention and diagnosis before the individual is clinically ill. Earlier diagnosis may result in long‐term benefits, including symptom‐free diagnosis, a reduction in DKA, a reduction in hospitalisation, and reduced misdiagnosis and mismanagement of type 2 diabetes (T2D) in adults [64, 68].

In addition to avoiding many of the worst outcomes associated with T1D, there is value for many individuals and caregivers to have time to prepare for a chronic disease [55]. Patients also may value being able to contribute to research to help others with the disease, including potentially their own family members, who are at high risk. Additionally, the stress for patients and caregivers is reduced with an earlier awareness of diagnosis in presymptomatic stages compared with a sudden and unexpected clinical diagnosis [68, 70, 71]. Screening also gives individuals the opportunity to discuss their results with and receive continuing education and monitoring from healthcare providers. An earlier diagnosis also gives individuals the opportunity to receive novel therapeutics that can alter the disease course [10, 64].

Screening initiatives are currently underway in areas such as the US, Europe, and Australia in high‐risk populations and in the general population [64]. Table 1 summarises the largest ongoing screening programs. Screening a larger pool of people—beyond those with a family history of T1D—with the goal of early detection and intervention, can potentially reduce long‐term healthcare costs associated with managing complications of undiagnosed autoimmune T1D. Large‐scale programs, such as the Combined Antibody Screening for Celiac and Diabetes Evaluation (CASCADE) programme in the US, use leftover samples from the Washington State Newborn Screening to test for genetic and islet autoantibody markers that estimate a child's risk of developing T1D and coeliac disease. The programme is free and does not require a blood draw or an appointment, but parents need to enrol [72]. The cost‐effectiveness of screening programs for large populations is being assessed. For example, Autoimmune Screening for Kids (ASK), an ongoing, large‐scale, presymptomatic, autoimmune T1D screening programme, assesses cost‐effectiveness as part of its outcomes [63, 73].

**TABLE 1:** Screening programs for T1D in the US, Europe and Australia.

Programme/site	Population screened	Location	Screening sites
Type 1 Diabetes TrialNet https://www.trialnet.org	2–45 years with first‐degree relative 2–20 years with any relative	International network	Sites across US, or an in‐home test
Combined Antibody Screening for Celiac and Diabetes Evaluation (CASCADE) https://cascadekids.org	0–8 months 4–8 years	Washington	Newborn screens and elementary schools
Population Level Estimate of type 1 Diabetes risk Genes in Children (PLEDGE) https://research.sanfordhealth.org/fields‐of‐research/diabetes/pledge	< 6 years 9–16 years	North and South Dakota, Minnesota, Nebraska, Iowa	Clinics and laboratories
Autoimmunity Screening for Kids (ASK) https://www.askhealth.org	1–17 years > 18 years	All of US	Sites across Colorado, or an in‐home test
Early Check T1D https://portal.earlycheck.org/	< 1 month	North Carolina	RTI International partnered with the University of North Carolina at Chapel Hill
Fr1da https://www.typ1diabetes‐frueherkennung.de	2–10 years	Germany	Sites across Germany
ELSA https://www.elsadiabetes.nhs.uk	3–13 years	UK	Schools, clinics or in‐home test
D1Ce (Pilot of the national screening programme) https://www.iss.it/‐/il‐progetto‐d1ce	2, 6, and 10 years	Italy	In‐home test
Global Platform for the Prevention of Autoimmune Diabetes (GPPAD) https://www.gppad.org/en/	< 1 month	Germany, UK, Poland, Belgium and Sweden	Clinics
ADIR	9–18 months and 5 years	Israel	Clinics
Type1Screen https://type1screen.org/whats‐involved	> 2 years	Australia	Sites across Australia or an in‐home test

Abbreviations: RTI, Research Triangle Institute; UK, United Kingdom; US, United States.

Although ongoing efforts are being made to identify individuals at risk for T1D development, it is crucial that all healthcare providers—primary care providers, pediatricians, and specialists—are aware of the risk for individuals with preexisting autoimmune disease or family history of T1D or other autoimmune diseases.

## 5 The DMT Era: Impact and Challenges

**FIGURE 2:**
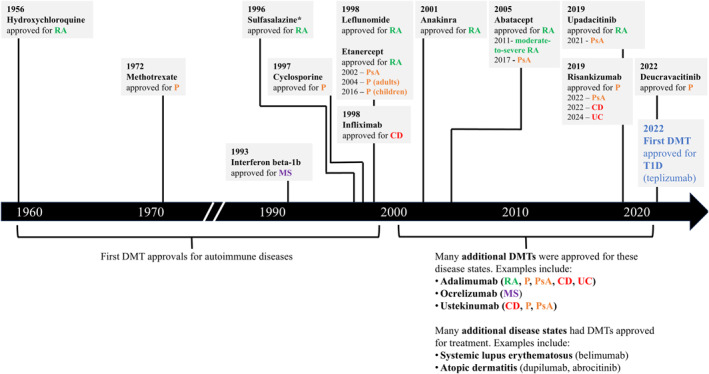
Select autoimmune diseases and timing of FDA approval of select DMTs. This figure shows select autoimmune disease states that have had DMTs approved for treatment over the past 60 years. In the past 20 years, the research and development of DMTs has drastically increased. Many additional disease states have introduced DMTs into treatment practices, and several new DMTs have been developed and approved for use. A comprehensive list of all autoimmune diseases and approved therapies is beyond the scope of this review. CD, Crohn's disease; DMT, disease‐modifying therapy; FDA, Food and Drug Administration; MS, multiple sclerosis; P, psoriasis; PsA, psoriatic arthritis; RA, rheumatoid arthritis; T1D, type 1 diabetes; UC, ulcerative colitis. *Originally introduced in the 1950s with positive clinical trials in the 1970s (oral suspension has since been discontinued).

Disease‐modifying antirheumatic drugs have been used for the treatment of rheumatoid arthritis since the early 1900s [2]. Since then, a paradigm shift in care has occurred in autoimmune disease treatment—with numerous DMTs approved for use in other autoimmune disorders, such as psoriasis, psoriatic arthritis, multiple sclerosis, and inflammatory bowel disease (Crohn's disease and ulcerative colitis; Figure 2). Even though these DMTs are not curative, several have become first‐line therapies in the treatment of these diseases and have been reported to provide significant benefit to individuals with these autoimmune conditions [74, 75, 76]. Studies have shown, for example, that DMTs substantially reduce the risk of disease progression in individuals with relapsing‐remitting multiple sclerosis and that individuals were satisfied with their treatment [77, 78]. DMTs have also been shown to reduce radiographic progression and disease activity in individuals with rheumatoid arthritis [79]. Individuals with moderate‐to‐severe plaque psoriasis reported being very satisfied with their disease‐modifying biological monotherapies [80]. The T1D field is now entering the era of DMT use and can use the lessons learnt from other autoimmune diseases as guidance on expectations, safety, and patient education [81].

**FIGURE 3:**
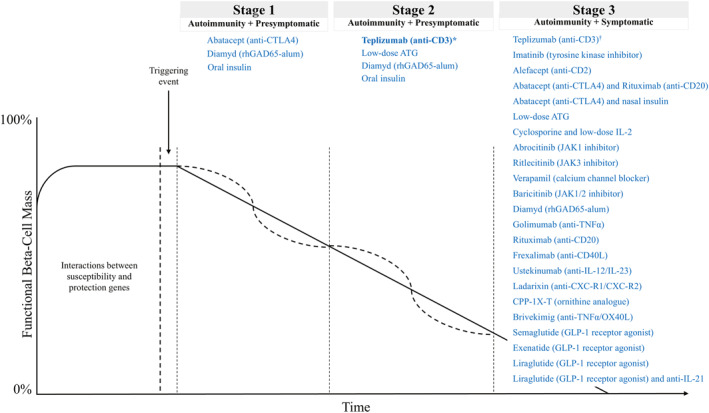
Eisenbarth curve showing the stages of T1D and DMTs that have been and are being investigated for use at each stage. The Eisenbarth curve is a model of the natural progression of T1D that depicts how functional beta‐cell mass is lost over time. This figure shows DMTs that have been and are currently being investigated for use in various stages of T1D. Beta‐cell regenerative therapies are also currently under investigation in individuals with clinical (stage 3) T1D. ATG, antithymocyte globulin; CD40L, CD40 ligand; CTLA4, cytotoxic T‐lymphocyte associated protein 4; CR, chemokine receptor; DMT, disease‐modifying therapy; FDA, Food and Drug Administration; GLP‐1, glucagon‐like peptide‐1; IL, interleukin; JAK, Janus kinase; OX40L, OX40 ligand; rhGAD65‐alum, recombinant human GAD65 conjugated to aluminium hydroxide; T1D, type 1 diabetes; TNF⍺, tumour necrosis factor alpha. *Teplizumab is the only immune therapy that has been approved for use in T1D by the FDA. It is approved for use in adult and paediatric patients ≥ 8 years old with stage 2 T1D to delay the onset of stage 3 T1D. ^†^As of publication, teplizumab has not been approved for stage 3 T1D in the US. *Source:* This figure is adapted from ‘Diabetes mellitus in childhood: an emerging condition in the 21st century’ by Della Manna et al. 2016 [82] and ‘Staging presymptomatic type 1 diabetes: a scientific statement of JDRF, the Endocrine Society, and the American Diabetes Association’ by Insel et al. 2015 [21], used under CC BY 4.0.

The natural history of T1D divided into stages, as first proposed by the late George Eisenbarth in 1986, depicts how functional beta‐cell mass is lost over time and helps inform strategies for therapeutic intervention to alter the disease course (Figure 3) [83, 84]. Teplizumab is an anti‐CD3 monoclonal antibody approved by the Food and Drug Administration for use in individuals with stage 2 T1D to delay the onset of stage 3 T1D. It has been shown to delay progression and the need for insulin by approximately 2–3 years compared with placebo (Figure 3) [3, 4]. The mechanism of action for teplizumab is not completely understood; however, by binding specifically to CD3 on T cells, it causes partial agonistic signalling and deactivation of the T cells that cause destruction of the pancreatic beta cells [85]. Specifically, it has been shown that treatment with teplizumab is associated with an increase in the proportion of T cells with an exhausted (deactivated) phenotype, which has been proposed to be a potential mechanism to reduce autoimmunity by rebalancing the immune system and has been demonstrated to preserve beta‐cell function as measured by stimulated C‐peptide in individuals with stage 3 T1D [4, 85]. Teplizumab has also been linked to an increase in the proportion of regulatory T cells (Tregs) in circulation [86], which appears to restore immune tolerance in the hyperactive autoreactive immune environment. Furthermore, teplizumab has been shown to promote operational tolerance, defined as a persistent effect following a single course of therapy. Specifically, a delay of stage 3 T1D onset of a median of 52.2 months was observed with one course of teplizumab compared to 27.3 months in the placebo group [87]. Safety was extensively investigated prior to FDA approval of teplizumab and is of utmost importance to physicians as they consider prescribing a new class of medication in T1D. The comprehensive safety experience with teplizumab was reviewed by Herold et al. in 2023 with data from 791 individuals treated with teplizumab representing ∼1500 patient‐years of follow‐up, and the safety profile of teplizumab was shown to be characterised by mild‐to‐moderate adverse events that typically occurred during or immediately after the treatment and were self‐limited. Higher rates of serious infections (teplizumab 3.5% vs. control 2%) were observed, although overall rates of infections were similar between treatment groups. Similar to what is seen with many DMTs, the majority of individuals treated with teplizumab (∼80%) developed transient lymphopenia, which resolved without treatment interruption [4]. Although not included in the integrated analysis, after an average follow‐up of 7 years, a subset of individuals treated with teplizumab showed no increased risk of infections and no malignancies [88].

The approval of teplizumab is likely to be the first of many approvals for DMTs since numerous therapeutics are being investigated for use in T1D (Figure 3). These therapies target various immune mediators that have been implicated in the pathogenesis of autoimmune diabetes, as well as the beta cells themselves. T‐cell‐targeting therapies include low‐dose antithymocyte globulin (ATG) and abatacept. Low‐dose ATG is thought to act by depleting pathogenic T cells. It has been shown to preserve C‐peptide levels and reduce glycated haemoglobin (HbA1c) in new‐onset stage 3 T1D [89, 90]. It is currently being evaluated for use to delay progression from stage 2 to stage 3 T1D [91]. Abatacept is a cytotoxic T‐lymphocyte‐associated protein (CTLA‐4) immunoglobulin that prevents T‐cell activation by blocking T‐cell costimulatory signals [92]. When given on its own to individuals with stage 1 T1D, the treatment was shown to help preserve insulin secretion but was not able to significantly delay the progression to stage 2 or stage 3 T1D [92]. However, clinical trials are investigating the use of abatacept in combination with other therapies, such as rituximab—the anti‐CD20 monoclonal antibody that selectively depletes B cells—and nasal insulin [5, 93, 94]. The effect of rituximab alone on beta‐cell function has also been assessed and it has been shown to delay the fall in C‐peptide levels and therefore preserve beta‐cell function in individuals with recent‐onset T1D [5]. A therapy involving the use of a short treatment of cyclosporine, followed by a low dose of IL‐2, is being investigated in individuals recently diagnosed with T1D [95]. Cyclosporine blocks effector T cells, while low‐dose IL‐2 activates and expands Tregs, which should restore immune homoeostasis [95]. In addition to targeting immune cells, therapies targeting cytokines have also been explored. For example, golimumab targets the proinflammatory cytokine tumour necrosis factor alpha (TNF‐alpha), which has been shown to play a role in beta‐cell destruction and death [6]. In children and young adults recently diagnosed with T1D, golimumab was demonstrated to preserve beta‐cell function [6]. Another therapeutic, brivekimig, targets TNF‐alpha as well as the T‐cell costimulatory molecule OX40 ligand and is currently being investigated in adolescents and adults with recently diagnosed T1D [96, 97]. The anti‐IL‐12/IL‐23 monoclonal antibody ustekinumab is another cytokine‐targeting therapy that has been shown to preserve C‐peptide levels in adolescents with recent‐onset T1D by inhibiting the generation of certain subsets of T cells [98]. Ladarixin, an inhibitor of the IL‐8 receptor C‐X‐C motif chemokine receptor 1/2, is also being investigated in adolescents with recent‐ onset T1D [99, 100]. The immune therapy, recombinant human GAD65 conjugated to aluminium hydroxide (rhGAD65‐alum), protects beta cells by inducing antigen‐specific immune tolerance to GAD65, one of the most common autoantigens associated with T1D [7]. It was shown to significantly affect C‐peptide retention in individuals newly diagnosed with T1D with a specific HLA genotype [7]. It is also being investigated for use in individuals with stage 1 and stage 2 T1D [101]. The Janus kinase 1 (JAK1) inhibitor abrocitinib reduces inflammation by preventing the activation of certain immune cells and is being investigated in a clinical trial to determine whether it can help preserve beta‐cell function in individuals newly diagnosed with T1D [102]. Lastly, frexalimab, the anti‐CD40 ligand monoclonal antibody, impairs the activation of innate and adaptive immune cells [103]. Its effect on endogenous insulin secretion is being investigated in individuals with newly diagnosed T1D [104, 105] (Figure 3).

Modelling studies have been conducted to compare the efficacies of these immune therapies. One study, for example, demonstrated that low‐dose ATG and teplizumab resulted in the highest levels of C‐peptide preservation at 1 and 2 years post‐treatment (low‐dose ATG: 55% and 103%; teplizumab: 48% and 63%, respectively) when compared with high‐dose ATG, rituximab, alefacept, abatacept, and low‐dose ATG/granulocyte‐colony stimulating factor [106]. Another study similarly demonstrated that teplizumab treatment resulted in a greater effect than abatacept, rituximab, and rhGAD65‐alum [107]. As we have observed in other disease states, individuals respond differently to different DMTs [31]. As more DMTs are approved, responder analyses are published, and clinicians become more familiar with these agents, a personalised medicine approach will be possible.

All the DMTs for T1D discussed thus far have focused exclusively on targeting components of the immune system. However, alternative strategies for modifying disease in T1D have also been explored, including using therapies that directly promote beta‐cell function and/or survival. Targeting the beta cells themselves is an important potential option for disease modification in T1D, and as a result, therapies that affect these pathways have been the subject of several clinical trials. Verapamil, for example, is a calcium channel blocker that acts on the beta cells directly to reduce beta‐cell apoptosis and has been shown to partially preserve C‐peptide levels in individuals with newly diagnosed T1D [108]. Baricitinib, a JAK1/JAK2 inhibitor, is another therapy that acts on beta cells directly. It is thought to prevent beta‐cell death by reducing major histocompatibility complex class I expression on islet cells, thereby blocking the interaction of pathogenic T cells with beta cells. Accordingly, baricitinib is an example of a drug that may influence both immune and metabolic pathways. It was shown to preserve beta‐cell function in individuals with newly diagnosed T1D [109]. Use of the tyrosine kinase inhibitor imatinib, which has been proposed to reduce apoptosis in beta cells by counteracting high levels of endoplasmic reticulum stress in the cells, has also been shown to preserve beta‐cell function in adults and was shown to improve estimated pancreatic beta‐cell function in a paediatric case study [110, 111]. The ornithine analogue CPP‐1X‐T is another therapeutic under investigation that has been proposed to act by reducing beta‐cell stress. Early studies suggest that this treatment has potential activity and may be well tolerated [112, 113, 114, 115]. Lastly, glucagon‐like peptide‐1 (GLP‐1) receptor agonists approved for helping to improve glycaemic control in T2D have also been proposed to reduce beta‐cell stress and apoptosis. As a result, therapeutics such as semaglutide, exenatide, and liraglutide have also been investigated for their ability to modify disease in T1D [116, 117, 118]. Liraglutide on its own as well as in combination with anti‐IL‐21 antibody has been shown to improve beta‐cell function (Figure 3) [118]. It will likely take a combination approach targeting multiple aspects of the immune system and beyond to achieve full clinical remission, which is the ultimate goal of immune therapy [31, 119, 120].

### 5.1 Potential for Clinical Remission in T1D

Beta‐cell preservation observed with the use of DMTs in individuals with new‐onset (stage 3) T1D may lead to clinical remission of T1D, which is defined as a reduction in the daily insulin doses required to achieve stable blood glucose levels shortly after beginning insulin treatment [121]. There are two main forms of clinical remission that can be achieved: partial and complete clinical remission [121]. Partial clinical remission defined by the International Society for Pediatric and Adolescent Diabetes (ISPAD) as an insulin requirement of < 0.5 U/kg/day and HbA1c < 7% (53 mmol/mol), and as an insulin dose‐adjusted HbA1c (IDAA1c) ≤ 9 by other groups, has been used as a clinical endpoint in several studies assessing the efficacy of immune therapies [121, 122, 123]. Complete clinical remission is achieved when no insulin treatment is needed for a period of time [121]. This form of remission is less studied, and spontaneous complete remission (i.e., without DMT) is less common [121]. Higher C‐peptide levels at clinical T1D diagnosis have been shown to be a factor that can influence the occurrence of both partial and complete remission [121]. However, both forms of remission are transient, and it has been proposed that using multiple therapies that target different disease pathways in a sequential manner will be necessary to achieve lasting remission [31, 119, 121]. To explore combinations of immune therapeutics with varying mechanisms of action, these therapies must first be approved as monotherapies, making the investigation and approval of novel immune‐targeting DMTs for T1D all the more important [119]. Additionally, in order to achieve a cure during the later stages of T1D, cellular therapies (including stem‐cell derived beta cells or islet transplantation) will likely be necessary to provide glucose‐sensing and insulin‐secreting cells to compensate for the limited beta‐cell function and mass at these late stages [124]. Preserving of beta‐cell function in stage 3 of T1D is also beneficial because it has been associated with fewer complications as the disease progresses and improved HbA1c and IDAA1c levels [50, 125, 126, 127].

### 5.2 Implementation of DMTs: Practical Issues

As mentioned, teplizumab—the first DMT for T1D—can now be considered for use in individuals with stage 2 T1D and has been shown to be efficacious and well tolerated [3, 85, 128]. However, since this DMT is the first in its class for T1D, most healthcare providers do not have experience and therefore comfort with this type of therapy [4]. Here, we will discuss in more detail the safety profile of teplizumab and other DMTs under investigation, as well as practical guidance for implementing these therapies in practice.

As mentioned previously, across multiple clinical trials in individuals with stage 2 or stage 3 T1D, common adverse events observed with teplizumab treatment included lymphopenia, rash, and headache. Most occurred during or immediately after the treatment and were resolved without intervention [4]. Cytokine release syndrome (CRS), an adverse event specific to immunomodulators that is described as a ‘constellation of clinical symptoms related to cytokine release’, was reported in 5.8% (46 of 791) and 1.2% (3 of 245) of individuals treated with teplizumab and control, respectively [4, 129]. Symptoms of this adverse event include fever, fatigue, headache, rash, joint pain, muscle pain, hypotension, hypoxia, and increased alanine aminotransferase, aspartate aminotransferase, and total bilirubin [130, 131, 132]. CRS typically occurs during the first 3–5 days of dosing and is resolved within 2–3 days of onset. Most events (88%) were grade 1 or 2 in severity and were treated with over the counter medications [4]. However, caution should be used when interpreting these data because the grading system used to evaluate CRS in these trials—the National Cancer Institute Common Terminology Criteria for Adverse Events—has been shown to be inconsistently applied by investigators [133]. Furthermore, since CRS is specific to immunomodulators, the number of these adverse events may have been under‐ or overreported by investigators who do not have much experience with these therapies. Awareness of the symptoms associated with CRS and the methods to mitigate this reaction are of clinical importance. Premedication of individuals with antipyretics (nonsteroidal anti‐inflammatory drugs or acetaminophen [paracetamol]), antihistamines, and/or antiemetics before each of the first 5 doses and as needed between subsequent doses of teplizumab can be done to help mitigate CRS [85, 130]. Proper adherence to the dosing regimen, which involves an initial small dose followed by gradually increasing doses, may also improve tolerability [85, 130]. Overall, the adverse events associated with teplizumab are primarily described as predictable, transient, and able to be mitigated. However, it is important to note that up to 15% of those treated in clinical trials were not able to tolerate a complete course of therapy. Therefore, it is expected that approximately 10% of individuals will not be able to tolerate a full course of teplizumab therapy in real‐world settings [4, 132]. The benefit after receiving an incomplete course of therapy is not well understood.

### 5.3 Safety Considerations With DMTs

As discussed, several other immune therapies and pathways are under investigation to modify T1D disease, including a selective costimulation modulator, a JAK inhibitor, a TNF‐alpha inhibitor, a CD40 ligand inhibitor, tyrosine kinase inhibition, and treatments targeting Th17 cells [6, 98, 104, 109, 134, 135]. For other therapies that have undergone clinical trials, safety profiles were also generally favourable. In small studies, no concerning safety signals have been revealed so far, and adverse events reported were similar between treatment and placebo groups [109, 134]. Hypoglycemic episodes were occasionally reported for some therapeutic trials in stage 3 T1D patients while on insulin, with some episodes reported as severe [134]. Serious adverse events reported are often not attributed to the treatment itself [109].

The risk of infection with these therapies has also been assessed. The rates of non‐serious infection observed in individuals treated with teplizumab and golimumab were similar in treatment and control groups, with an infection rate of 53.0% in the group treated with teplizumab and 52.7% in the control group specifically for the integrated analysis of safety in individuals with stage 2 and stage 3 T1D [4, 6]. For the teplizumab study, higher rates of serious infections were observed in the teplizumab group (3.5%) compared with the placebo group (2%) [4]. In the 7‐year follow‐up study, rates of mild respiratory and other infectious complications were similar for individuals treated with teplizumab (*n* = 31) and those treated with placebo (*n* = 12) [88]. Additionally, during a study evaluating the use of teplizumab in individuals with newly diagnosed T1D, which took place during the COVID‐19 pandemic, the incidence of COVID‐19 was similar between teplizumab and placebo, and COVID‐19‐related adverse events were not increased with teplizumab [123, 136]. For the golimumab study, no serious infections were reported [6]. More data are needed on the longer‐term risk of infection, but it is expected that the risk with teplizumab will be low because the lymphopenia observed was brief, typically occurring in the initial ramp‐up period, and resolved while dosing was continued [4]. This is being evaluated in a long‐term safety registry [137].

As clinicians consider the risk‐benefit ratio when prescribing any medication, it is important to note that individuals with symptomatic T1D who do not receive DMTs are potentially at higher risk for long‐term complications with lower C‐peptides, some of which have been discussed [127]. Individuals with clinical T1D also have a greater susceptibility to infections and have worse outcomes from infection [138, 139]. In one retrospective matched cohort study comparing individuals with and without diabetes, almost 15% of individuals with clinical T1D had an infection requiring hospitalisation, which was more than 3 times the risk in individuals without diabetes [139]. Individuals with clinical diabetes also experienced a higher risk of developing kidney infection (3.0‐ to 4.9‐fold), osteomyelitis (4.4‐ to 15.7‐fold), and foot infection (6.0‐ to 14.7‐fold) compared with individuals without diabetes [138]. Lastly, individuals with clinical diabetes experienced a 2‐fold higher rate of death from COVID‐19 [138]. Therefore, the concern about infection related to immune therapy must be viewed in the context of the risk of poor outcomes when patients are not offered DMTs.

Although DMTs generally involve the use of injections or infusions, doses are often given intermittently. Teplizumab, for example, requires only a single 14‐day course of treatment to observe an approximately 2‐year delay of disease onset versus placebo [85]. Longer‐term studies need to be performed to determine the duration of the effect of teplizumab and whether this dose needs to be repeated or another agent offered—and, if so, the ideal timing needs to be understood.

### 5.4 Future Direction of Immune Therapies

DMT treatment of individuals with T1D has shown varied responses, with some responding to treatment while others not [4, 6]. It has been proposed that this difference in response to therapy is due to the presence of distinct T1D endotypes, T1D subtypes that exhibit distinctive biological or functional features, in individuals with the disease. Each endotype is the result of differences in the immune and nonimmune networks that drive T1D pathophysiology present among individuals [140]. How these differences relate to different therapeutic responses is not fully understood. Therefore, an important future direction for the use of DMTs in T1D is to continue to investigate and characterise these different disease subtypes and to identify biomarkers associated with response to therapy. This will allow for more personalised treatment options to individuals, which may have a greater chance of success [31]. The development of more targeted therapies, such as those that are antigen‐specific designed to induce tolerance in T1D, will be a major benefit to the field since it could potentially provide disease prevention or long‐lasting drug‐free remission without global immunosuppression. Investigations into other protein‐based, peptide‐based, cellular, and even nanoparticle‐based approaches are underway. Additional research into the timeline of T‐cell reactivity to these antigens over the disease course of T1D will be important for these approaches [141].

Beta‐cell replacement therapies that use stem‐cell‐derived, fully differentiated, insulin‐producing islet cells are in phase 1/2 clinical trials [142, 143, 144, 145]. Insulin‐secreting organoids, in vitro three‐dimensional structures that are also derived from stem cells, are emerging as another potential future beta‐cell replacement therapy. Safety concerns regarding organoids remain to be resolved and efforts to optimise the techniques necessary for the use of this therapy continue to evolve, meaning that this therapeutic approach may become a reality in the future. Future research will focus on optimising cells used for derivation and immune‐evasive strategies as well as confirming long‐term safety and efficacy in large‐scale clinical trials [146].

Widespread use of beta‐cell replacement therapy, however, may be limited since it currently requires chronic immunosuppression [145]. As a result, several beta‐cell regenerative therapies are also being investigated as alternatives, where the pancreas regenerates its own beta cells [145]. An example of this type of therapy involves the use of stem cells [147]. Additional endogenous beta‐cell regenerative therapies in early development include BMF‐219, which is thought to drive the proliferation, reactivation, and preservation of beta cells by inhibiting the protein menin, and an oral triple therapy that combines gamma‐aminobutyric acid, a dipeptidyl peptidase‐4 inhibitor, and a proton‐pump inhibitor [148, 149, 150]. There are other beta‐cell regenerative therapies in the early phases of development [151]. These therapies are promising, but additional clinical trials are necessary to optimise and validate these treatments and to address the challenges associated with these therapies, including biological stability and safety [145, 147, 149].

Identifying effective DMTs for T1D has proven complex. As discussed previously, although teplizumab has become available, there is some consensus that combination DMTs may provide additional clinical benefit. Numerous strategies for improving the speed and efficiency of trial design have been proposed, including adaptive designs with key early biomarkers as intermediate endpoints, factorial designs of combination DMTs, and use of master protocols in well‐defined T1D risk cohorts. Combinations of DMTs with synergistic mechanisms—those that have positive impacts on beta‐cell function coupled with therapies targeted at mitigating autoimmune T‐cell responses—are of strong interest. Therapies that target other immune cells (e.g., B cells and antigen‐presenting cells) and therapies that protect, restore, and/or replenish beta cells are of interest as well to test in combination [120].

## 6 Conclusion

T1D is the result of chronic autoimmune‐mediated destruction of insulin‐producing pancreatic beta cells. With the approval of the first DMT for T1D, the focus of treatment is shifting from treating T1D as a metabolic disorder to modifying the underlying immunology to preserve beta cells and therefore endogenous insulin production. Immune therapies are necessary for this approach and have been shown to be efficacious in slowing the decline of beta‐cell function, thereby enabling patients to live without symptomatic disease for longer periods of time and, in some patients, prevent progression to the need for insulin [3, 87]. Additionally, these therapies have shown acceptable safety profiles to support ongoing clinical development, finally ushering in an era of DMT use in T1D similar to other autoimmune diseases. Once more therapies are approved for clinical use, the next stage is studying combination therapies to improve beta‐cell preservation and incorporate personalised medicine into T1D care.

## Author Contributions

C.D., P.A.G., A.W.M. and B.N. conceived of the review and critically reviewed and edited the manuscript. All authors have read and approved the final manuscript.

## Conflicts of Interest

C.D. has lectured for or been involved as an advisor to Viela Bio, Provention Bio, Sanofi, Amarna, SAB Therapeutics, AstraZeneca, Shoreline Bio, Immunocore, Quell, and Vertex. He holds a patent jointly with Midatech plc and Provention Bio/Sanofi. P.A.G. is a co‐founder, CMO and shareholder in IM Therapeutics, Inc. He has received grant support from NIH, Helmsley, JDRF, Nova Pharmaceuticals, Intrexon T1D Partners, Sanofi, Biomea, Imcyse, and Provention Bio. He has consulted for Provention Bio, Viacyte, Imcyse, Sanofi, JDRF T1D Fund, Cour, GentiBio, Abata, and SAB. A.W.M. is a co‐founder and shareholder in Immunomolecular Therapeutics and has participated in a Sanofi data safety monitoring board or advisory board. B.N. has participated in Sanofi advisory boards and Sanofi speaker bureaus.

## Peer Review

The peer review history for this article is available at https://www.webofscience.com/api/gateway/wos/peer-review/10.1002/dmrr.70091.

## Data Availability

Data sharing not applicable to this article as no datasets were generated or analysed during the current study.
